# Integrating Reconfigurable Hardware-Based Grid for High Performance Computing

**DOI:** 10.1155/2015/272536

**Published:** 2015-03-22

**Authors:** Julio Dondo Gazzano, Francisco Sanchez Molina, Fernando Rincon, Juan Carlos López

**Affiliations:** Escuela Superior de Informatica, Universidad de Castilla-La Mancha, 13071 Ciudad Real, Spain

## Abstract

FPGAs have shown several characteristics that make them very attractive for high performance computing (HPC). The impressive speed-up factors that they are able to achieve, the reduced power consumption, and the easiness and flexibility of the design process with fast iterations between consecutive versions are examples of benefits obtained with their use. However, there are still some difficulties when using reconfigurable platforms as accelerator that need to be addressed: the need of an in-depth application study to identify potential acceleration, the lack of tools for the deployment of computational problems in distributed hardware platforms, and the low portability of components, among others. This work proposes a complete grid infrastructure for distributed high performance computing based on dynamically reconfigurable FPGAs. Besides, a set of services designed to facilitate the application deployment is described. An example application and a comparison with other hardware and software implementations are shown. Experimental results show that the proposed architecture offers encouraging advantages for deployment of high performance distributed applications simplifying development process.

## 1. Introduction

Scientific community is continuously increasing the demand for HPC [1]. Due to its nature, high performance applications are adapted to the existing computing capacity in order to achieve a good tradeoff between accuracy and processing time. Therefore, a system intended to provide a faster and accurate solution in HPC turns out of great interest [2].

FPGA-based scientific computation is one of the solutions for scientific community to improve the response time for numerically intensive computation [3]. Approaches for high performance reconfigurable computing (HPRC) integrate both processors and FPGAs into a parallel architecture. HPRC can achieve several orders of magnitude improvement in speed, size, and cost over conventional supercomputers [4, 5]. FPGAs offer high performance close to ASICs but, in contrast to these, FPGAs provide a high degree of flexibility similar to a general purpose computer. FPGA-based systems are faster than a pure software approach in terms of computational power [6]; therefore, an infrastructure allowing for the exploitation of the high performance capability of a hardware implementation with the benefits of autonomous resource management is a promising alternative to be used in HPC.

However, the process needed to accelerate applications using reconfigurable hardware has required so far some expertise in hardware design in order to obtain maximum benefits from the hardware platform where the design is being implemented. People that use HPC mostly design their application by means of high-level languages such C, C++, but mapping an application onto hardware resources requires to describe it using hardware description languages (HDL). Although there are tools that help designers to obtain HDL code from C or C++ source code [7–9], to maximize the benefits of using hardware, it is necessary to have a good knowledge of hardware design. These benefits (speed, power, etc.) also depend on the target FPGA vendor. Furthermore, each FPGA vendor offers a set of features, such as primitives or dedicated hardware (DSP, processors, etc.), which help to obtain better results for specific architectures.

Besides the gap between Sw and Hw programming concepts that makes translation to hardware challenging, there are some aspects that have so far prevented the use of reconfigurable hardware-based platforms in HPC: the lack of tools for deployment of computational problems in a hardware platform usually leading to ad hoc structures, the lack of configurable or parameterizable hardware resources, and the lack of debugging methodologies and the low portability of components (unlike in software where the portability is high with the use of libraries), among others [10].

One way of bringing reconfigurable hardware-based platforms closer to HPC users is to develop a platform and a set of tools oriented to facilitate application partition and deployment and the integration and communication of heterogeneous components and to provide a complete platform management service that contemplate users and resources. In this regard, this work proposes the necessary infrastructure for the management and use of reconfigurable computational resources and the interfaces that are needed to obtain a system as generic as possible, increasing the possibilities of developers when creating compatible applications. Besides, a set of services provided to attain transparent user-application deployment has been developed. One of the key aspects is the use of the dynamic reconfiguration capabilities of FPGAs. Using dynamic reconfiguration allows for fine-grain modification of FPGA functionality, maintaining a set of services in the FPGA and changing only the area dedicated to user applications [11]. This work also provides the infrastructure for an efficient use or the partial reconfiguration capability of FPGAs.

This paper is structured as follows: related works are summarized in the next section; then a brief description of the architectural view of the platform is presented in Section 3. In Section 4, the infrastructure management system is presented where basic administration services are described. Next, in Section 5, the architecture computing nodes are defined, and partial reconfiguration issues are discussed. In Section 6, an example reconfigurable application design is detailed. Later, in Section 7, experimental results are presented and discussed. Finally, the last section presents conclusions.

## 2. Related Works

High performance computing clusters have evolved towards the inclusion of different kinds of hardware accelerators in order to overcome the limitations in the amount of parallelism achievable with commodity CPUs. Heterogeneous clustering is more efficient than homogeneous architectures, and tightly coupled accelerators help to reduce communication requirements by making use of data locality [12].

From the architectural point of view, accelerators can be included in the cluster using two different approaches: either as uniform node nonuniform system (UNNS) or as nonuniform node uniform systems (NNUS) [4]. In the first case, each node in the clusters includes only a single type of resources (i.e., only CPUs, GPUs, or FPGAs). Examples of this configuration are the SGI Altix servers [13], Netezza for data warehouse applications [14], the Convey HC1 and HC-1ex hybrid computers [15], or the Cray XD1 [16], just to name a few of them. In some cases, FPGA technology is hidden behind an extended instruction where designated operations are accelerated in the hardware fabric. In other cases, FPGAs can only be accessed through a tightly coupled processor using a closed API. The main drawback of this approach is the communication overhead between processing elements (PE) and the need of special hardware for the integration of the accelerators with the communication backbone, which has a high impact in the final cost of the solution. In the NNUS approach, nodes are composed by a mixture of different kind of processing elements, but all of them include a similar set of characteristics, thus providing a homogeneous view of the cluster. One important advantage of the model is that it can take benefit of high-speed interconnection links between local PEs, providing much higher communication bandwidth without requiring special-purpose communications hardware. Also, the NNUS architecture is better suited for the single program multiple data (SPMD) paradigm, where a single copy of the program can be easily scaled to a multinode cluster.

Examples of NNUS nodes are the Axel [12] architecture or the commercial FPGA-based computer RYVIERA and COPACABAN platforms. SCIEngines [17] provides developers with a bare reconfigurable platform in which FPGAs resources are at the same level of processors. However, the development environment is not trivial for nonexpert hardware personnel.

The kind of systems described in the introductory paragraphs of this section falls into the category of the so-called heterogeneous computing platforms. Heterogeneous computing platforms are now in the heart of servers for HPC since the fall of single CPU clusters and the replacement of multicore-based systems. In the following, we disclose some of the most relevant works and existing technology for each one of the two main working areas where this work will contribute to the state of the art, namely, (1) FPGA integration techniques and (2) development model and tools.

### 2.1. Contributions to FPGA Integration Techniques

There are several reasons to integrate FPGAs also in HPC. The first and most obvious reason is performance. Secondly, and equally important, is the low power consumption. Finally, the last reason is the flexibility obtained when using FPGA in contrast to other hardware acceleration strategies used in HPC [18]. Most of the solutions in this concern place FPGAs as a simple coprocessor of a master entity (i.e., an on-board CPU) that typically runs a control program. FPGAs are, thus, surrogated to a lower level, behind the processor. This is the dominant role of FPGAs both in high performance embedded systems [19–23] and HPC servers.

Normally, this is done via FPGA PCI-Card solution, expanding the traditional computational node, and a producer-consumer computational model. This architectural solution allows easy technology adoption, but it has some drawbacks such as the communication overhead between hardware and software.

Examples of this configuration are [13–15] or [16], among others. In some cases, FPGA technology is hidden behind an extended instruction set where designated operations are accelerated in the hardware fabric. In other cases, FPGAs can only be accessed through a tightly coupled processor using a closed API.

Most of works using FPGA for HPC repeat the strategy of integrating an accelerator into applications to speed up the execution of the kernel of an algorithm [24, 25]. Nevertheless, this strategy is not intended to execute the whole application in hardware.

The approach presented in [26] represents an evolution with respect to the acceleration of a single algorithm. It offers an architecture where reprogrammable hardware resources can be used as if they were resources managed by the operating system, abstracting in this way user applications.

The proposed architecture is based on a card with partially reconfigurable FPGAs connected to the bus of a general purpose computer. This architecture loads those hardware components needed to accelerate an application, through a software layer that incorporates these FPGAs as if they were additional system resources. This work does not provide hardware communication transparency and replication services.

There are other proposals that use dynamic reconfiguration and on-the-fly bitstream relocation as the main features for the employed architecture. Into this group, one can find [27], where dynamically reconfigurable FPGAs are included. In this approach, reconfigurable areas are used to load application accelerators behaving as slaves. This fine grain reconfiguration allows a better use of resources and the possibility to launch more than one hardware core at the same time in the same FPGA. An inconvenient matter about this model is that communication from software to hardware provokes a reduction of performance when the hardware computation time is similar to the reconfiguration time. This work does not provide communication mechanisms between cards, limiting parallelization.

The Erlangen slot machine architecture [28] belongs to this group as well and proposes a system that exploits dynamic reconfiguration and relocation of bitstream to implement high performance AV stream applications. Their advantages are deployment transparency and communication inside the FPGA, but this architecture was not designed to scale to more than one device.

Another strategy used to accelerate an algorithm is the use of soft-core processors implemented in FPGAs, as those processors created using Mitrion C software [29, 30], which are customized and replicated automatically for each application. With this strategy, an increment of performance of the algorithm execution is obtained, but it is limited by Sw-Hw communication mechanisms. Its main advantage is related to application development due to the fact that developers use C-like software abstracting them from hardware details.

Most of these related works keep users tied to the physical architecture (Figure 12), so, the effort to take advantage of architecture specific resources needs to be done again when the architecture change (low portability). One solution is to use a logical architecture in order to provide designers with the same architecture, abstracting them from physical architecture and providing also the infrastructure to perform mapping efficiently from logical to physical architectures. The main idea is to present an abstraction from computational resources to users, in order to keep them as far as possible from the underlying infrastructure, avoiding having a deep knowledge of the platform to perform an efficient application deployment.

### 2.2. Contributions to Development Models and Tools

One of the main problems with heterogeneous PEs integration is the very different nature of their architectures, which also affects the programming model typically used in each case. Regarding GPUs, considerable efforts have been done by vendors and the scientific community to provide high-level programming models and frameworks, such as CUDA [31] from Nvidia or, lately, the recent announcement of Altera [32] that uses OpenCL as the unique programming model for FPGAs, GPUs, and CPUs, and, as a result, they have been quickly adopted by mainstream software programmers. However, that is not exactly the case for FPGAs.

While FPGAs have proven to be very power efficient and good candidates for HPC, particularly for applications demanding fine-grain parallelism and nonstandard data sets, they have important drawbacks: they require highly specialized design skills, vendor toolchains are too focused on the optimization of the resulting circuits, but they provide little support for seamless integration of the solutions, and last but not least portability is still a challenge, even for different FPGA families of the same vendor. The research community has been working in high-level synthesis tools for more than a decade, in order to close the enormous productivity gap for FPGA design, and recently the release of several of these tools (such as Catapult-C [33] from Calypto, Vivado HLS [34] from Xilinx, or Synphony [35] from Synopsys) is beginning to show good expectative. However, these tools only address the problem of the hardware implementation of computational kernels, while there is still a long road to turn reconfigurable logic into a resource commodity.

Current state of the art in synthesis tools technology provides support for a big subset of the C language, excluding those aspects that cannot be easily mapped to a hardware implementation, such as dynamic memory management. Also the coding style and mapping rules have been simplified, broadening the community of users and not just targeting engineers with a hardware design background. It is only required to understand some basic concepts related to how compilers work. Most of these tools accept a plain C, C++, and SystemC description that can be shaped into a hardware implementation despite the definition of certain directives, such as the type of protocol to define the reception of the arguments, loop manipulation, or the identification of blocks that may benefit from a parallel implementation. The whole process of directive definition, synthesis, and results analysis takes no more than a few minutes, providing a mean to quickly explore the design space, which is in contrast to the several weeks that the same task would require following classic hardware design flows.

## 3. Model

The progress and expansion of distributed high performance application in software have been tied to computation platform development and their development models. These platforms have relied on (a) distributed systems technology that contributes to an interconnection middleware between clients and services, (b) object oriented modeling, providing design advantages through the data and functionality encapsulation, and (c) the movement toward clusters and grids facilitating the pooling and exploitation of computational resources.

An important aspect of each existing platform relies on its model. A model allows developers to take advantage of heterogeneous resources. The model will provide flexibility and tools, enabling the designer to develop applications. The model also gives information about the types of the existing computing elements, their interconnections, and their performance. The success of a platform is tied to the facility and the accuracy with which a real problem can be modeled or represented. Then, to facilitate the deployment of a distributed high performance computation problem, the adopted model must provide the advantages already provided by software approaches plus the benefits offered by reconfigurable hardware:

- problem modeling facilities;
- automatic deployment;
- location transparency;
- communication transparency;
- replication mechanisms.

Besides, the adopted model must specify several service levels depending on the expected application control degree, offering lower barriers and development facilities to both sides of HPC business: users and service providers. Furthermore, the adopted model should provide flexibility to dynamically compose available resources in order to optimally exploit the computational platform for a specific computational problem.

The platform model proposal presented in this paper, that we call reconfigurable grid (R-Grid), identifies a component model in the foundations of the solution to explore. This model will be supported by the physical platform and it will be the basement for application analysis, modelling, refactoring, and development.

The R-Grid platform model will ease the way application development is nowadays performed in FPGAs, particularly parallel applications, by means of this component model and domain specific libraries. The concept of component is technology independent. This makes the R-Grid proposal portable and not attached to the existing technology, allowing for the evolution of the concepts and techniques result of this research, to further and future technology generations.

In our approach, an application is constituted by a collection of components, whose nature depends on the adopted programming model and the chosen target hardware resource. The R-Grid platform model will facilitate and promote the use of the distributed reconfigurable computation resources through domain specific primitives and components. In domains where artifacts as primitives and domain specific components already exist, they will be efficiently ported to the new specific hardware. In other cases, they will be inferred from the analysis of certain use cases and will be included in an available library.

R-Grid platform model supports different programming models such as those based on shared memory (openMP), remote method invocation (RMI), or messages passing interface (MPI).

## 4. Architecture Description

The infrastructure proposed in this paper gives the necessary support for transparent application deployment and automatic application management, in order to liberate users from architectural aspects during runtime.

This infrastructure was designed in three layers: the lowest layer is composed of reconfigurable resources on which user applications are instantiated; the second layer abstracts these resources providing a resource homogenization and facilitating application deployment; and the upper layer is used for resource and application management issues.

From a top-down perspective, the basic architecture of the platform comprises a central* management node* (upper layer) and a set of interconnected reconfigurable* computational nodes* with their corresponding communication adapters (second and lower layers). The management node is implemented in software and offers a set of basic services for resources and application management that will be described in detail later.

Computational nodes, on the other side, offer computational capacity (resources) and are composed of two main parts: one part consisting of a set of resources dedicated to accommodate user applications, and the other part includes a resource abstraction creating a homogeneous view to facilitate node integration.

These computational nodes can be either software nodes (processors) or hardware nodes (reconfigurable logic). Software computational nodes are built on a general purpose processor where applications are deployed in software, while hardware computational nodes are FPGAs with dynamically reconfigurable areas, where applications are implemented in hardware.

**Figure 1:**
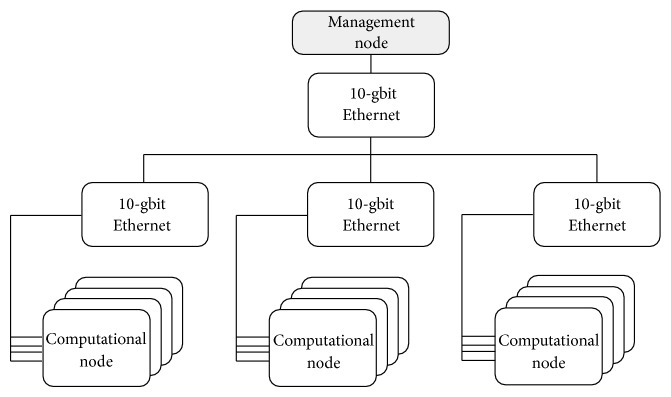
Infrastructure composed of several computational nodes managed by a management node.

A scheme of the described infrastructure is depicted in Figure 1.

Both computational nodes and management nodes are interconnected through a high performance network. To obtain a homogeneous view of resources, a communication abstraction model based on the OOCE middleware [36] was used, where the communication between nodes is performed using remote method invocation (RMI). Through RMI, it is also possible to manage the deployment of applications as binary files, as well as to obtain information about the computational node type, the state of resources, and so forth. The OOCE middleware facilitates component adaptability and the communication between components using the client-server approach as it will be described later. For each client component, a proxy is added that represents the server component. If the client requires services from different servers a proxy for each one will be added to the client. Each proxy has the same interface as the correspondent server. Proxies translate the invocation sent to the servers into messages through the communication channel. In turn, a skeleton is added to each servant object in order to translate messages into invocations to the servant.

### 4.1. Management Node: The Infrastructure Management

The implementation of the management node is purely software. The technology used during development process was as follows: JAVA was used as a programming language, OOCE used as communication middleware, JDBC for database access, and SQLite as database.

Platform management aspects are covered by a set of basic services offered by the management node. These services are a key part of the system and are defined to facilitate the exploitation of computational resources in a simple and transparent way. These services are essential to facilitate both the deployment of applications and the use of the reconfigurable platform. The following subsections describe each one of these basic services.

#### 4.1.1. Application Registry Service

The application registry service is the first service that a user employs.

This service allows the clients to manage their application repository. The repository is the only source of application supported by R-Grid so clients as a previous step to execution must include their application in the repository. The repository stores structural and binary information of applications.

Before delving into the application registry service, it is necessary to briefly describe the application model that better fits with the proposed infrastructure. This application model is intended to meet those requirements that can appear due to the use of not only software computational nodes but also dynamic reconfigurable hardware resources.

**Figure 2:**
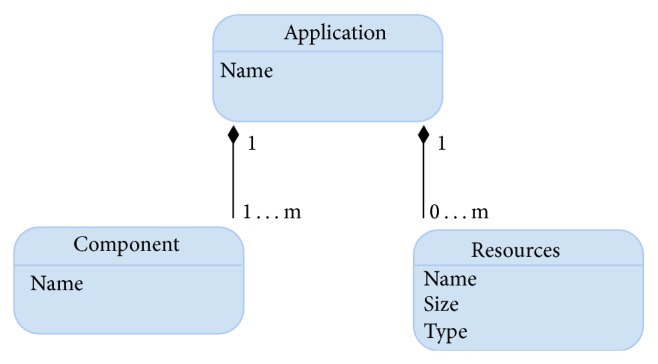
Application model.

Applications can be seen as a set of components interacting with each other to reach the solution of a certain problem using specific resources (Figure 2).

**Figure 3:**
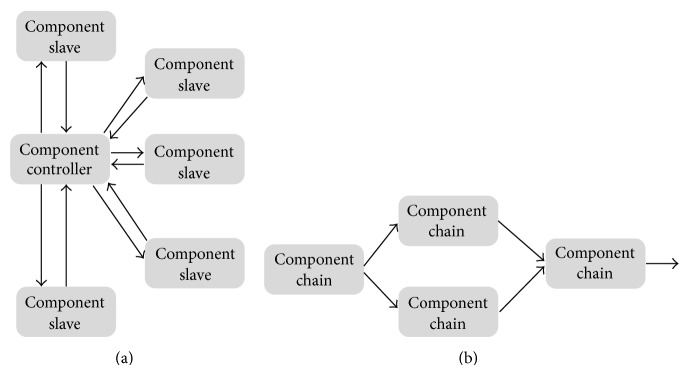
Applications schemes.

Each component is defined as a programmable unit to be instantiated in a computational node resource. Examples of application configurations are shown in Figure 3.  Figure 3(a) describes an application composed by a controller interacting with several slave components. In Figure 3(b), an example of application composed by chained components is shown.

The application structural information contains data about the name of the application, its components, and the required resources defined in the application model. The set of all this information is called* application descriptor*.

The application registry service is in charge of collecting information about the application composition, that is, the application identifier, the components of the application, and the component binary files associated with a specific computational node model. To select the computational node model that better fits with the application to be deployed, the management node offers a list of computational nodes with different characteristics (i.e., memory bandwidth, area, maximum clock frequency, etc.) to the users. Users select the specific computational node and generate the corresponding binary file for the chosen resource. The application identifier must be unique for each user. Likewise, each component has a unique component identifier in order to be reached by the remaining components of the application, independently of their locations.

#### 4.1.2. Application Deployment Service

Once the application has been registered, the user can request the deployment of the application.

The deployment request triggers the loading of the binary file associated with the selected resource model contained in a computational node already registered in the previous step.

Besides the fact that the binary files have been created for a specific type of resource, the management node uses a set of metrics to select the proper computational node between all those that can hold the same bitstream for the given application constraints (bandwidth, memory, etc.). For example, in order to maximize the bandwidth, the objective of the management node is to deploy all components in the same computational node (i.e., in the same FPGA). Once the computational node has been selected, a binary file is transferred for resource configuration.

#### 4.1.3. Application Location Service

As stated before, applications are defined as a set of collaborative components that can be instantiated in different distributed resources using several computational nodes. Then, to have a correct application execution, a transparent communication between local and remote application components is necessary.

To facilitate the access to local and/or remote components, the application location service provides location information for the application components. This is a hierarchical service composed of a global locator running in the management node and a local locator implemented in each FPGA. The global locator keeps location information about all components of an application that are deployed in the system, while local locator keeps information of only locally deployed components (in the same FPGA or processor). Assuming that the location of a component can vary during the lifetime of the application depending on the priority of use of free resources, may occur that a component need to be moved either inside the same computational node or to another FPGA, in order to comply with some application restrictions (i.e., performance). Each time a component is deployed, the location service is updated. Then, if a component that has been moved needs to be reached, the request is redirected to the local locator first to obtain the new local address of the component and in case the component has been moved to a different FPGA the request is then redirected to the global locator which is provided with the new component address. This indirection process occurs only when a component changes its location. Once the component location is identified, the corresponding proxy is updated for further invocations. The location service uses a data structure that includes a component identifier as a primary key and the content associated with that key is the physical address of the component.

#### 4.1.4. Data Structure Implementation

Data structure is a key part of management node. The management node will receive a plethora of concurrent request and has to maintain all information about system state in a safe and coherent way. The structure of data has been implemented through a relational database and a file system. The database will keep the dynamic information of system while the file system will store the application binary files.

**Figure 4:**
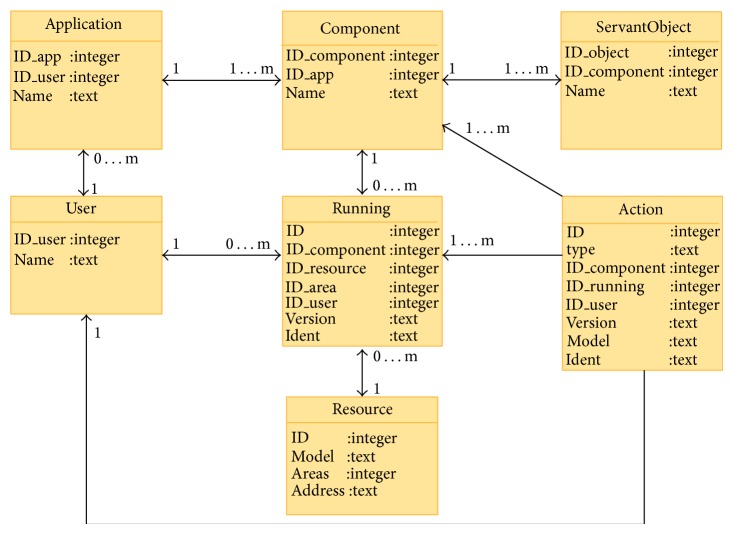
Database relational diagram.

The dynamic information of the system will include registered users, their applications, the state of each application, the pending actions to be performed (activation, stop), and the existent resources and their states. All this information follows the scheme represented in Figure 4.

### 4.2. Computational Node: The Resources Infrastructure

Computational nodes have two objectives. The first one is to offer their internal resources to the management node for application deployment. The second objective is to provide a homogeneous view independently of their nature (hardware or software), in order to allow the management node to have a common treatment for all of them independently of their model. The only requirement is that all computational node models must offer the same interface.

**Figure 5:**
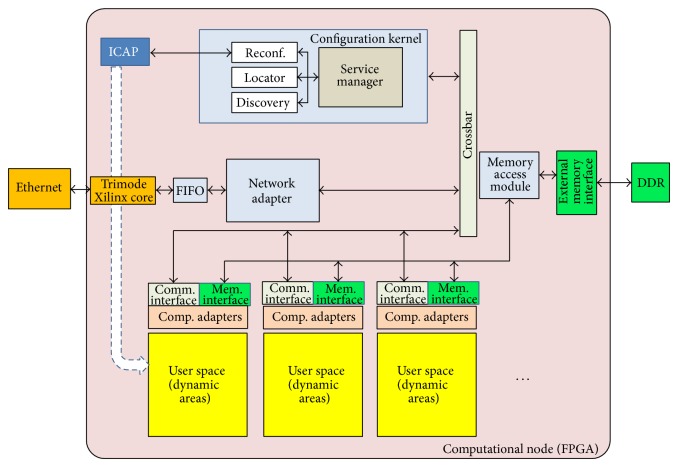
Computational node architecture.

When hardware, the computational node can be formed by one or by a set of reconfigurable areas, depending on the FPGA model. Each one of these areas, named dynamic area (Figure 5), is a resource that can be configured to contain a functional component of the user application. In case of dynamically reconfigurable FPGAs, all reconfigurable areas inside the same FPGA are defined of the same size and with the same amount of internal resources. This allows for the relocation of an instantiated component from one dynamic area to a different one in the same computational node or relocated in a different FPGA of similar characteristic. A scheme of the computational node is depicted in Figure 5. The amount and size of dynamic areas depend on the size and type of FPGAs; therefore, the architectural model, presented in this paper, combines computational resources of different characteristics in order to offer a wide range of reconfigurable resources for application deployment. Users can choose specific resources for their application selecting them from those offered by the management node as detailed in Section 4.1.

Dynamic areas have two interfaces, one dedicated to communicate either with the configuration kernel or with the rest of components belonging to the same application and the other one dedicated to provide access to local memory. The component named memory access interface allows the sharing of memory resources between all dynamic areas dividing space memory into spaces of local memory for each dynamic area separately.

The configuration of each dynamic area is performed by the configuration kernel component. This component provides three kinds of different services as follows:

- reconfiguration service which is in charge of partial bitstream management during application deployment; this service is invoked only by the management node when a user requires application deployment;
- location service that is part of the application location service provided by the management node as described in Section 4.1; this is an internal service whose main objective is to accelerate internal location consulting; this service is used to locate a component inside the computational node;
- self-discovery service that allows FPGA to be discovered by the management node with the startup of the FPGA.

Self-discovery service is a key functionality for resource management. When connected, each FPGA will announce their own features (model, size), amount of resources, and type of areas to place components. This announcement is performed during the startup of the FPGA and the execution of the three described services is managed by the service manager component. It provides an interface offering methods such as* get_device_model*,* get_device_free_resources*, and* deploy_bistream*, to configure resources. Through this interface, the management node can query each configuration kernel component of each FPGA about the computational node model or about the amount of free resources to launch a reconfiguration process in a specific dynamic area (Figure 16).

The network adapter component, in turn, translates the incoming messages into invocations to the configuration kernel or to the components placed in dynamic areas and composes internal invocations into messages to the outside.

#### 4.2.1. Communication Model

One of the main characteristics of the grid systems is the possibility of task relocation according to several factors such as the amount of free resources, the policy employed in task distribution, or specific user requirements among others. To facilitate portability of components between different computational node models, a common communication interface (resource abstraction) is provided by the platform. As briefly introduced in Section 4, the communication model used in this work is based on the OOCE middleware. When using OOCE middleware, each component is the sum of both the functional core plus the corresponding adapters, proxy, or skeleton, depending on the type of functional core. In this way, a client will be formed by the client core plus the proxy of the server that will attend the clients invocation, and the server will be formed by the server core plus the skeleton. The proxy will translate the invocation to the communication channel protocol and, on the other side, the skeleton will translate from communication channel protocol to server invocations. Using this communication mechanism, the invocation between two remote components can be seen as a local invocation. This allows components to be adapted to the corresponding communication infrastructure isolating the functionality from the communication channel. The generation of these adapters to the corresponding communication channel is performed through the use of R-Grid tools from a description of the corresponding component interface.

This approach allows three degrees of transparency: (a) location transparency, because the client sees the server interface as if it was a local invocation, (b) access transparency because it is possible to reach the server independently of its implementation (hardware or software), and (c) communication transparency because this middleware can be used for any communication channel.

#### 4.2.2. Network Protocol Requisites

Classical cluster system generally uses network protocols such as TCP/IP, but this is a stack that is not easy to implement in the hardware. For that reason, a specific protocol based on TCP/IP has been developed for the R-Grid infrastructure. This protocol should comply with a set of requisites in order to have a very easy hardware implementation and with a very low communication overhead.

- Global system addressing: the first requisite in R-Grid is to have a transparent communication between components independently of the nature of the computational node they are placed.
- Orthogonal communication: the second requisite is to facilitate the access to either the component of an application or to the system services, using the same message format for both cases.
- Bidirectional communication: the third requisite is to have a bidirectional communication allowing the return of a value or a confirmation of completed operation or error messages.
- Safe communication: the protocol should also provide security communication issues in order to prevent the access to applications that do not belong to the corresponding user or the access of components from one application to another one.
- Broadcast communication: the protocol should provide broadcast messages.

**Figure 6:**
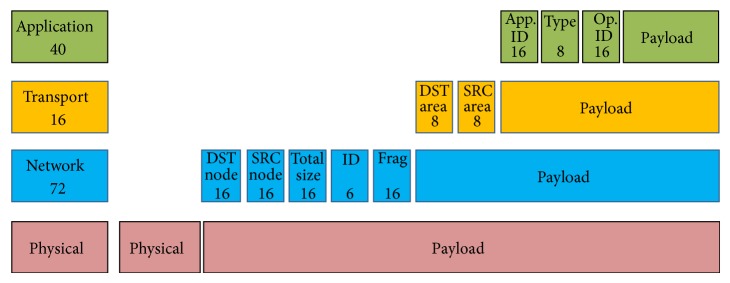
Network protocol layers.

Starting from these requisites, the network protocol will be divided into a set of layers as depicted in Figure 6. These layers and the fields that each layer introduces into the message are described as follows.

*Physical Layer*. This layer makes upper layers independent of transmission media. Physical media used in* R-Grid* go from* Ethernet* till point to point communication* RocketIO*, or* SATA*. For* Ethernet* the physical layer has a field with source and destination* MAC* address plus a field to identify type of message.

*Network Layer*. This layer implements addressing functionality. It will be used to transport messages from a specific computational node to another inside the system.

 
*DST Node*. The* DST node* field indicates the destination computational node. It uses three special addresses that will not change in any* R-Grid* system:
  - address 0 indicating its own node;
  - first assignable address for the management node;
  - last assignable address for broadcasting messages.
 
*SRC Node*. Equivalent to* DST node* field, this field stores information about the source of the message.
 
*Total Size.* It indicates the size of the* payload* in this layer.
 
*ID*. It allows the identification of a message between different transactions. It is used to deal with message fragmentation.
 
*Frag.* This field contains information for fragmentation process control.

*Transport Layer*. This layer implements the multiplexing of functionalities inside computational node allowing hoosing a service or a deployed application component.

 
*DST Area*. The* DST area* field indicates which component in the computational node is the destination of the message. It uses two special addresses:
  - address (0) indicates services zone in the computational node;
  - the last assignable address indicates all deployed components.
 
*SRC Area.* The* DST area* field stores information about source component.

*Application Layer*. This layer implements information about operations and type of message.

 
*App. ID*. This field provides the application ID. This value is unique for each deployed user's application. All components of the same application will have the same ID. This field allows verification if a message received by a component belongs to the same application.
 
*Type.* It indicates if the message is a shipment, a reception, or an exception.
 
*Op. ID.* This field indicates the operation or functionality of the invoked component. If the functionality is a service, this field indicates the specific required service. The list of the operations corresponding to the platform services is described in Table 1.

### 4.3. Partial Reconfiguration Infrastructure

The reconfiguration process starts when the management node sends an invocation to the configuration kernel to deploy a bitstream. The configuration kernel uses the internal configuration mechanism of FPGAs. Xilinx FPGAs have a component named internal configuration access port (ICAP) to perform the configuration of a partial region of the FPGA. The ICAP provides access to the FPGA configuration interface and the configuration registers performing a partial reconfiguration of the FPGA. The configuration kernel has been designed to deal with the internal reconfiguration process of the ICAP. The configuration kernel acting as a dedicated DMA is an asynchronous component that reads the bitstream from memory and transfers it to the ICAP. This is done with burst transactions, allowing for fast partial reconfiguration of the FPGA without intervention of a processor. The ICAP receives the bitstream from the configuration kernel and loads it into the configuration register of the FPGA. This is the only component in the proposed infrastructure that is technologically dependent.

Besides, the configuration kernel updates the location information with valid component endpoints in the location service when necessary.

As dynamic areas in the same FPGA are defined with the same shape and size, the bitstream can be modified to be used in different areas [37]. The resources in terms of logic involved in the implementation of the configuration kernel and time overhead during partial bitstream reconfiguration are evaluated in Section 6.

## 5. Designing a Reconfigurable Application

The application design is determined mainly by its functionality, which is divided into small pieces and programmed in several components. Each one of these components is assigned to a* dynamic area*. Then, an application is composed of a set of partial bitstreams where each partial bitstream represents a component of the application.

A component consists of the functional core itself plus two interfaces. One interface is named communication interface and performs the communication to the rest of the application. The second interface is a proxy to memory, a memory interface.

The memory interface is a generic technology that is an independent description of the capabilities of a memory: read and write operations of a single word or a sequence of words. Such a description is a logical representation of real memories in the system. Any memory block in the system can be modeled with the memory interface and used by means of its proxy, while, from the implementation point of view, we are simply reading and writing to a certain address computed from a base (the proxy reference) plus an offset, the address specified in the methods.

Components can have a passive role, and that means the component is waiting for an incoming invocation to process data and return a result or can have an active role invoking other components.

*R-Grid* offers to application developers a lot of freedom in modeling their application architecture. The two basic rules are the explicit intertask communication and the distributed memory. In R-Grid architecture hardware tasks can communicate each other without the action of a host; this avoids host-coprocessor architecture bottleneck existing in some HPRCs.

Another advantage is that developers can implement and deploy their code without restrictions because each task runs in a hardware sandbox. This freedom is compatible with a library of implemented common tasks. In this way, the developer can choose tasks from library or implement their own design.

**Figure 7:**
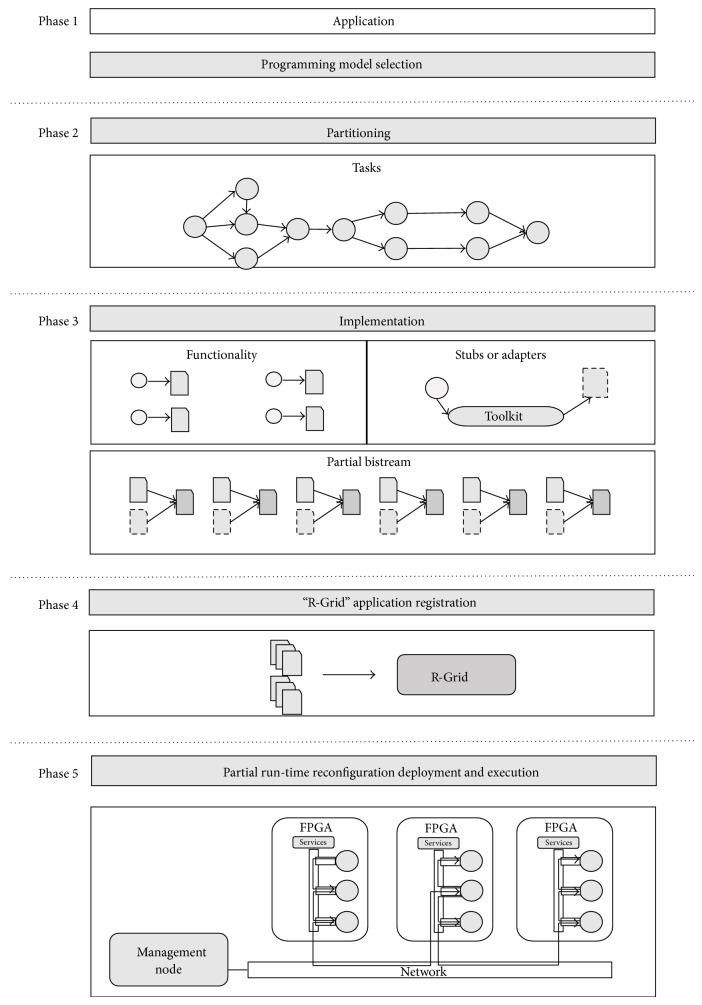
Application development flow.

In Figure 7, we can see briefly the application development workflow proposed by R-Grid.

It has five phases from the applications analysis to the application execution. The first phase corresponds to the selection of the programming model. Once the application has been designed according to the selected programming model, the next step is the partition of the application into a set of cores as subtasks. The next phase has two parts, one consisting in the generation of the corresponding stubs or adapters to form the component (core plus adapter) that will be implemented in hardware, plus the generation of the corresponding partial bitstream. These bitstreams will be generated according to the model of the selected FPGA. In this stage, the application descriptor is created. In Phase 4, the management node comes into the game when application and binary files are registered. Finally, the management node deploys the application in R-Grid architecture.

The synthesis process and the creation of the binaries files are performed using the tools provided by FPGA vendors. For computational nodes based on Xilinx FPGAs, we used Xilinx tools with support of dynamic reconfiguration. In order to simplify the synthesis process, R-Grid is provided with a project template containing constraint files, communication interfaces, and the element forming the static part of the FPGA, plus the initial partial bitstreams.

In order to choose an example that can reflect the needs of most scientific applications like those requiring solutions of several numerical linear algebra problems, such as eigenvalue problems, linear least square problems, and linear system of equations, among others [38], a matrix multiplier application is chosen as an example to be implemented. This example has two objectives: the first one is to illustrate how a high performance application is implemented in this infrastructure. The second objective is to test the platform.

This matrix multiplier is based on three functional cores: the data controller, the multiplier, and data storage. The* data controller* is in charge of matrix partitioning to perform calculus in parallel. The controller divides the multiplication process by the number of multiplier components involved.* Multipliers* are in charge of calculating partial results of the matrix partition assigned by the data controller. Finally, the* data storage* functional core integrates partial results to create the result matrix.

Once the data controller, multiplier, and data storage functional cores have been developed, it is necessary to add both of the network and memory interfaces to each functional core to create the corresponding component.

As stated before, interfaces (proxies and skeletons) to the communication channel and to the local memory are automatically generated using R-Grid tools.

To synthesize each component, a project template is provided that includes partial bitstream of target FPGA. The template is copied for each component of the application. In each copy of the template, the functionality source code and the autogenerated adapters code of the corresponding component are included forming each final component that will be instantiated in the computational node. Finally, a TOP design including designed components is included. Once components have been generated, they are synthesized and bitstreams are generated. Components are ready to be instantiated into dynamic areas.

The next step is the creation of the application descriptor in order to proceed with the application registry in R-Grid. The application descriptor is described in Algorithm 1 (Application Descriptor example).

Those bitstreams are loaded by the configuration kernel in the specified dynamic areas.

After application has been deployed, data controller assigns the corresponding data to be processed to the local memory associated with each multiplier deployed. After completion, the data controller sends an invocation to each multiplier component to start the multiplication process.

## 6. Experiments and Analysis

To test and validate the infrastructure and the described services presented in this work, several experiments have been developed.

### 6.1. Computational Node Implementation Analysis

The analysis of the implementation of a computational node has the following objectives: characterization of communication in terms of logical resources used in communication mechanism and available bandwidth; analysis of deployment time; and the characterization of location process in terms of involved resources and the time consumed in location process.

#### 6.1.1. Characterization of Communication

Table 2 shows the amount of resources involved in the communication mechanism of the computational node in a Virtex 5 FX70T FPGA.

**Figure 8:**
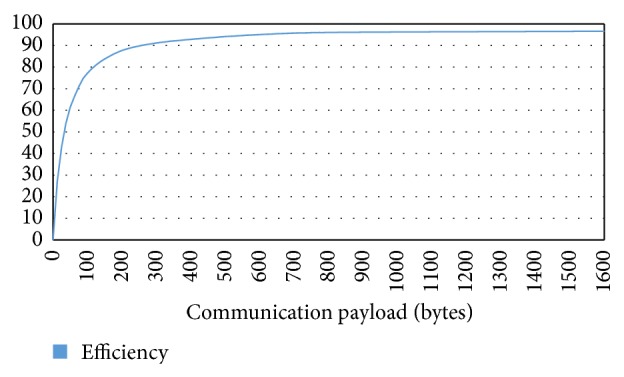
Efficiency of R-Grid protocol.

**Figure 9:**
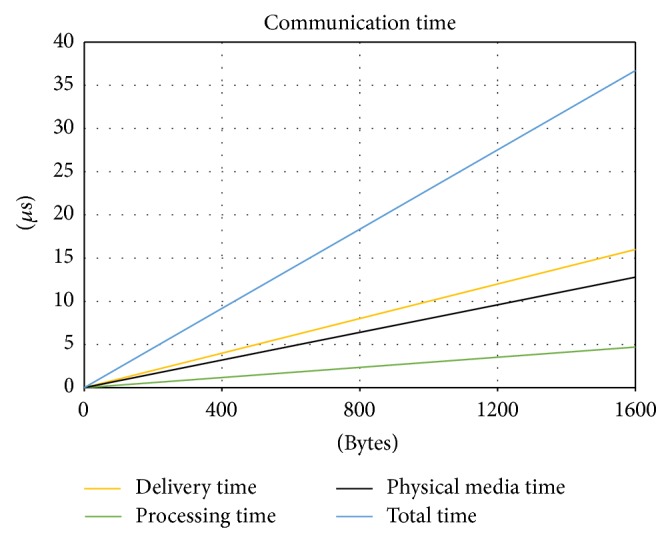
Communication time in each part of the communication process.

Regarding communication performance, the first analysis was the evaluation of the efficiency of R-Grid protocol. The results are depicted in Figure 8 with respect to the size of communication packets. In the graph, it can be observed that the header of the message has low overhead from 90 bytes, reaching an efficiency of 75%.  Figure 9 shows the time consumed during different size packets transmission in each part of the communication mechanism of the computational node. The reception and transmission time (delivery time) represent the minimum time obtained if the network could satisfy the internal bandwidth. Processing time represents the time consumed in packet processing in a component performing echo function. Physical media time represents the transmission time for a 1 Gb Ethernet network. Total time includes reception, transmission, and processing time. As can be observed, the network communication time is a limiting factor to deliver data between remote processing elements.

#### 6.1.2. Characterization of the Deployment Process

**Figure 10:**
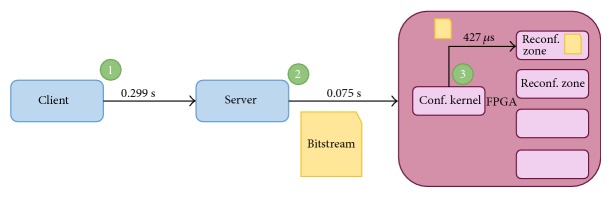
Reconfiguration time.

This test case has the objective of evaluating the reconfiguration time using R-Grid configuration kernel. In Figure 10, the involved blocks during deployment process and the time consumed in the reconfiguration of a 130 KB size bitstream, measured at each involved stage: request, bitstream transference from server to computational node, and computational node configuration, can be seen.

Table 3 shows the amount of resources involved in the deployment process.

#### 6.1.3. Characterization of the Location Process

This test case was intended to evaluate the time consumed in the location process described in Section 4.1.3. The test consists in the location process of components *A* and *B* requested by component *C*. In this test, component *A* is placed in the same computational node of component *C* while component *B* is placed in a different node in order to evaluate local and global location process, respectively.  Table 4 summarizes the time consumed in both cases. In the first case, the location service is provided by the local locator whereas the global locator is consulted in the second one.

### 6.2. Matrix Multiplication: SPMD and RMI Programing Model

The next experiment consists in the implementation of a matrix multiplier in the platform to analyze the viability of the proposal, evaluating whether the services are correctly implemented and the benefits are obtained in the complete application deployment process. This experiment was performed following the steps indicated next. First, a programming model for application development was chosen. Then, an analytical study about application needs and the necessary FPGA capabilities in terms of amount of logic, memory capacity, and bandwidth was done in order to check viability of the selected resources. The next step is to implement the application and the evaluation of resources. The last step consisted of evaluating whether the performance obtained is competitive compared with a traditional software solution.

The matrix multiplication example was developed following the SPMD (single process, multiple data) technique, using RMI as communication mechanism, and distributed memory.

**Figure 11:**
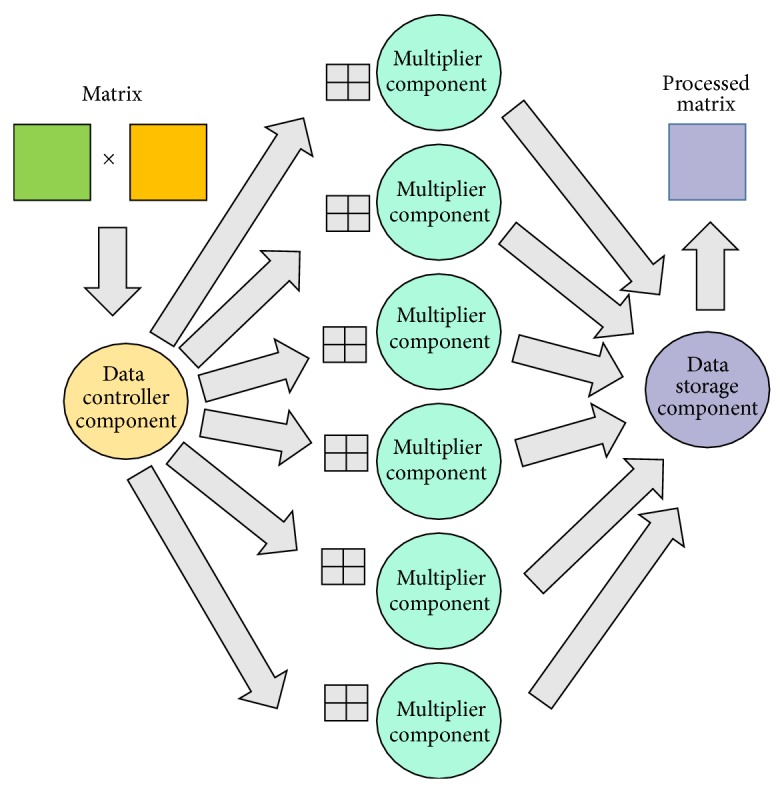
Logical architecture defined for the experiment. These three roles compose the logical architecture where the number of processing elements (multipliers) is variable.

**Figure 12:**
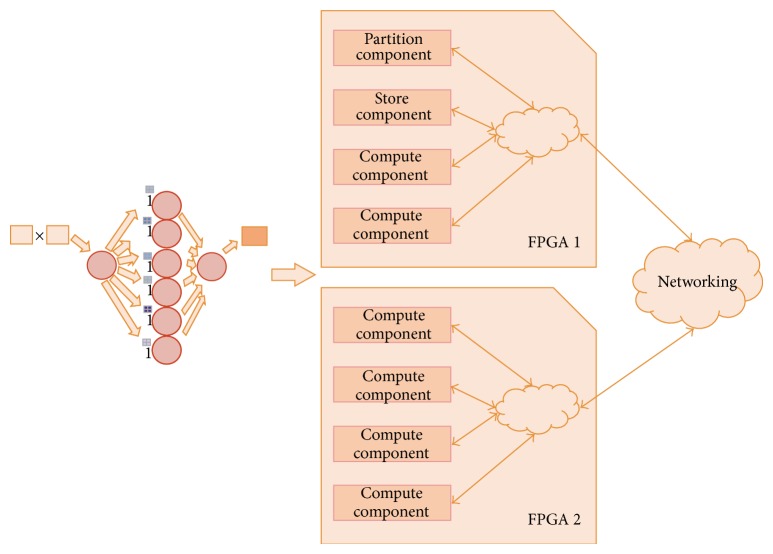
Physical architecture.

A simple application architecture was proposed where three main roles were defined (see Figure 11).

- Data controller role: this role is responsible for data reception. Once received, data is partitioned and sent to computing elements. In this experiment, the data controller role was implemented in the data controller component.
- Processor role: this role is responsible for data processing. This role can be played by several components placed in one or several nodes (multiplier components). Once data are processed, they are sent to the next player.
- Data storage role: it receives the processed data and it is responsible for combining the final data from the received partial data. This role is performed by the data storage component.

#### 6.2.1. Data Controller Role

This role has several methods as indicated in Algorithm 2 ( Data Controller Role description).

The first action is to store each operand matrix. This is performed through* stoAArray* and* stoBArray* methods, where int* m*, int* p,* and int* n* parameters indicate matrix dimension (rows and columns). Data partitioning is coordinated through* Calculate* method. This method is invoked indicating the amount of processing elements that will be used for matrix multiplication defined by the* ProcessingElements* parameter.

*M*, *P*, *N*, and ProcessingElements parameters are used to determine submatrix partitioning process.

#### 6.2.2. Processor Role

Processing role was divided into two parts. One is devoted to store partial data received from data controller and the other is for multiplication process. The* stoSubMatrix* method stores partial data in local memory starting from the memory address indicated by* pos* parameter. The parameters* sizeXp* indicates the total amount of submatrix elements, and* Number_of_vectors* indicates the number of data vector sent to the processing element, whereas* data* parameter indicates the value of the corresponding matrix element (see Algorithm 3 Processor Role description).

To start multiplication process, data controller invokes* multSubMatrix* method where* posi* and* posj* parameters denote the position of each operand (already given by the* stoSubMatrix* method), the* size* denotes how many times the operation must be done, and the last two parameters* resultX* and* resultY* indicate the position, in the result matrix, where the result of the multiplication must be saved.

#### 6.2.3. Storage Role

The storage role has methods for result matrix initialization, for obtaining data, and for data writing (see Algorithm 4 Storage Role description).

### 6.3. Physical Capacities Evaluation

As hardware devices two Xilinx FPGAs were selected: Virtex 5 VLX110T and VLX220T, with a DDR2 Micron MT8HTF12864HDZ-800 memory, 1 GB of capacity, and a peak transfer rate of 6400 MB/s, the model of the application corresponds to that shown in Figure 3(a), where the controller distributes matrix operand elements to computational kernels to perform the calculus in parallel. For that in each FPGA, four components were implemented.

#### 6.3.1. Logic Requirements

Matrix multiplication is defined in (1), where *A* is a *M* × *P* matrix, *B* is a *P* × *N* matrix, and *C* is a *M* × *N* matrix:

(1) $$\begin{array}{c} A\times B=C,\\ C_{ij}={\left[AB\right]}_{ij}=\overset{p}{\underset{k=1}{\sum }}A_{ik}*B_{kj}. \end{array}$$

To define the size of reconfigurable area for matrix multiplier component implementation, we start developing a parameterizable matrix multiplier component. This component is a computational kernel that solves a *Q* × *Q* submatrix of the whole matrix of results. The computational kernel can solve *Q* × *Q* values per cycle. Input data are the *Q* rows and *Q* columns of the original matrix operands.

The computational kernel was implemented using 1 × 1, 2 × 2, 3 × 3, and 4 × 4 submatrix sizes. These implementations were performed in two versions: one using DSP macros and the other without DSP macros.

Dynamic areas were defined using Xilinx PlanAhead tool. Each area occupies one clock region following vendor's advice. Both FPGA models have 16 different clock regions; therefore, each dynamic area offers 1/16 part of total resources. In this experiment, eight reconfigurable areas were defined to implement up to 8 components.

**Figure 13:**
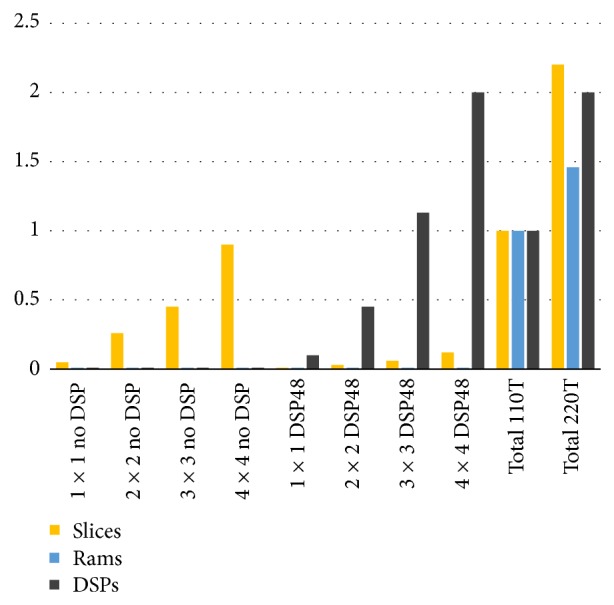
Logic resources requirement (normalized 110T).

The synthesis data report is summarized in Figure 13, where* Total 110T* and* Total 220T* represent the total of available resources (normalized to 110t) per dynamic area defined in the* Virtex 5 VLX110T* and the* VLX220T* models, respectively. In this graphic, it can be observed that all computational kernel configurations can be implemented in defined dynamic areas, except 3 × 3 and 4 × 4 model using DSP in Virtex 5 VLX110T,

Now, the reconfiguration time is evaluated. The reconfiguration time depends on partial bitstream size and the reconfiguration latency.

The configuration kernel includes a hardware FIFO and it is optimized for burst reads and writes from the DDR memory to the ICAP controller. Virtex 5 ICAP controller supports 32 bits/clock cycle bandwidth. As a result, the latency does not suffer from the memory* I/O* bottleneck and is completely delimited by the ICAP reprogramming latency; thus, it is near the technological limit of the device. In this experiment, the size of each partial bitstream for Virtex 5 110T FPGA model is of 233 KB and the reconfiguration time using configuration kernel is 596.5 *μ* sec.

#### 6.3.2. Memory Bandwidth

The second analysis carried out was to determine whether the available memory resources are sufficient for each computational kernel. For this, it is necessary to calculate the amount of memory and the memory bandwidth needed by each model of the computational kernel.

Memory bandwidth is defined by the kernel size, and by the matrix element size, as it can be observed in the following equation:

(2) $$\begin{array}{c} \text{Bandwidth}=\text{Kerne}{\text{l}}_{\text{size}}*\text{Dat}{\text{a}}_{\text{width}}*2. \end{array}$$

**Figure 14:**
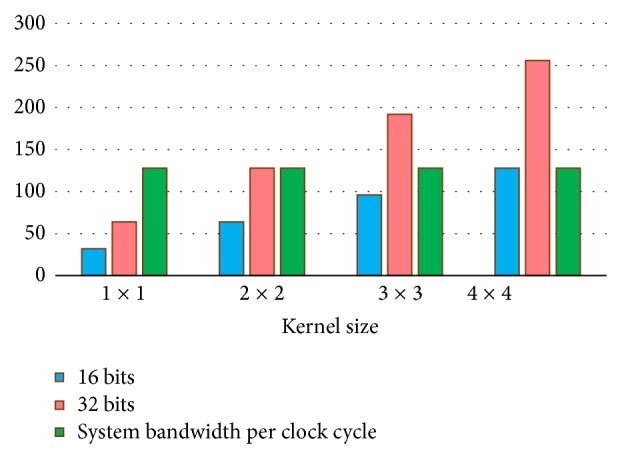
Memory bandwidth requirements per cycle.

Figure 14 represents memory bandwidth requirements for 1 × 1, 2 × 2, 3 × 3, and 4 × 4 kernel size, for two sizes of matrix element: 16 bits and 32 bits. As a reference, the total memory bandwidth supported by Virtex 5 was represented.

As it can be observed for a 16-bit-wide matrix element, the memory bandwidth is sufficient for all sizes of computational kernel, being optimum 4 × 4 computational kernel. For a 32-bit-wide of matrix elements, only 1 × 1 or 2 × 2 computational kernel can be used; otherwise, the memory bandwidth is not enough to feed the computational needs of the kernel.

#### 6.3.3. Memory Capacity

The need of storage space for the resultant matrix depends on the size of matrix operands, the width of matrix element, and the size of the kernel adopted. The memory space for the resultant matrix is defined by *M*_total_ while the memory space reserved for component operation is defined by *M*_component_ just the way it is described in formula (3), where *M*, *P*, and *N* represent matrix dimensions:

(3) $$\begin{array}{c} M_{\text{total}}=\left(M*P+P*N+M*N\right)*\text{Dat}{\text{a}}_{\text{width}},\\ M_{\text{component}}=\left(2*P*\text{Kerne}{\text{l}}_{\text{size}}\right)*\text{Dat}{\text{a}}_{\text{width}}. \end{array}$$

**Figure 15:**
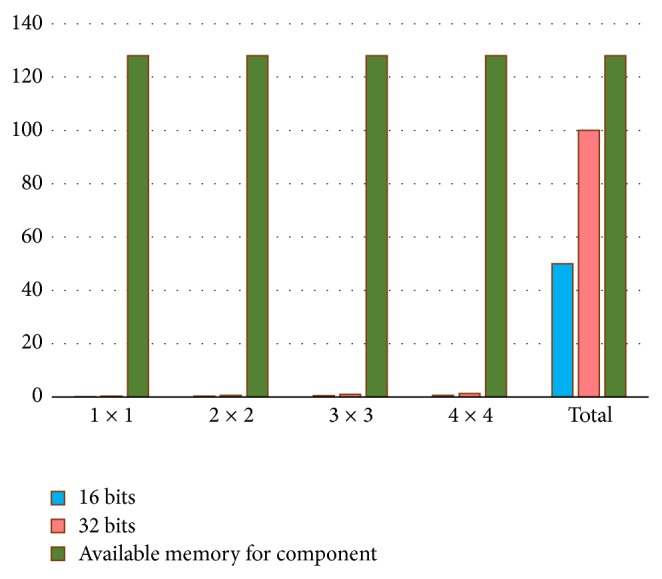
Storage requirements (matrix size 5000 × 5000).

**Figure 16:**
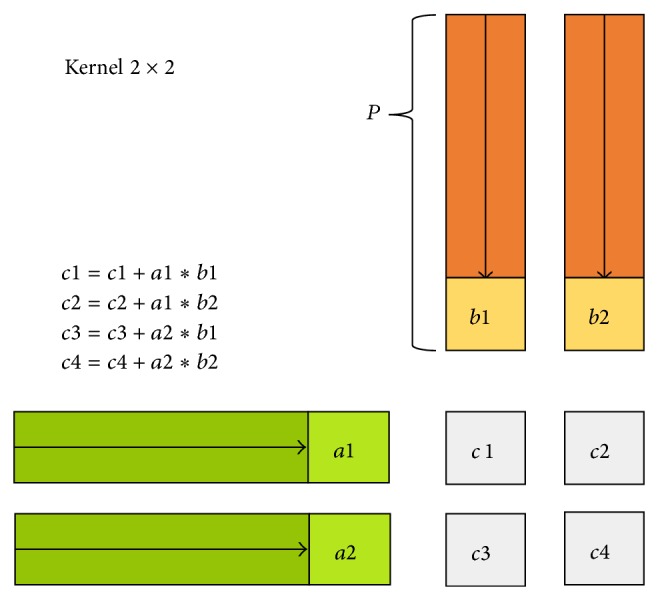
Kernel 2 × 2 composition.

Figure 15 represents the memory capacity requirements with respect to kernel size. For each component, 128 MB of memory space was reserved, allowing for the storage of the whole matrix.

### 6.4. Performance Evaluation

**Figure 17:**
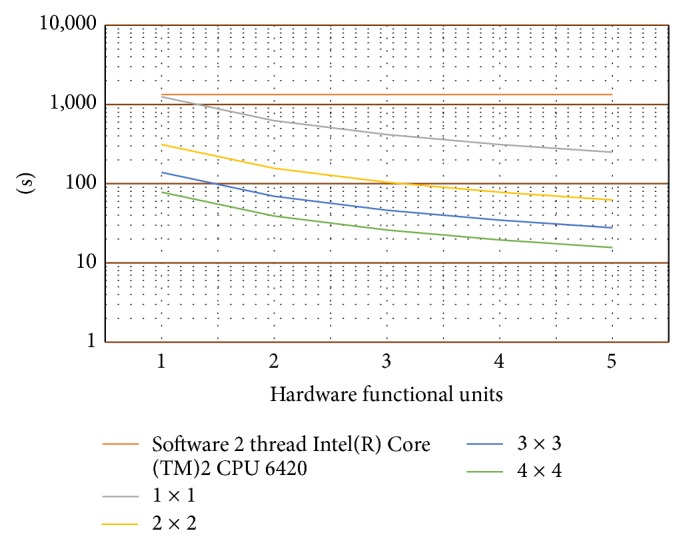
Time to compute (matrix 5000 × 5000 16 bits).

Once the memory space was defined, the next stage consists in performance evaluation for a 5000 × 5000 16-bit-width elements matrix multiplication, taking into account the computational kernel size and the amount of replicated computational units used. The result can be observed in Figure 17. This graphic shows time consumed (in seconds) during matrix multiplication using several hardware approaches (different kernel size) and a software solution.

Computation time needed by one kernel is determined by the size of the original matrix and the size of the kernel. Each 1 × 1 kernel can perform a multiplication of one column element and one row element and the result is added with the accumulated value in the kernel in one clock cycle. Then, the total time, in clock cycles, consumed is equal to the number of columns of matrix *A* or the number of rows of matrix *B*. Once this time is known, the next step is to determine how many times this operation needs to be done, as it is defined in formula (4), where *M*, *P*, and *N* represent matrix dimensions:

(4) $$\begin{array}{c} \text{Tim}{\text{e}}_{\text{component}}=P,\\ \text{Tim}{\text{e}}_{\text{total}}=P*\frac{M*N}{\text{Kerne}{\text{l}}_{\text{size}}^{2}}. \end{array}$$

**Figure 18:**
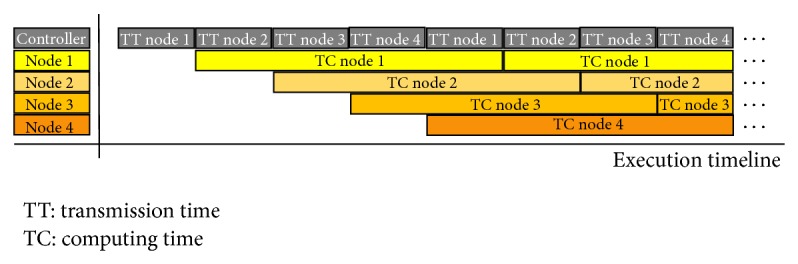
Timing diagram.

In order to attain an execution time very close to the computation time calculated above, it is necessary to hide the data transference time with the computation time of each computational kernel. In order to optimize the execution time and to avoid that the computational kernel remains inactive until new data is loaded in its local memory, it is necessary to determine the minimum amount of data to be loaded into local memory to ensure continuous processing time. It is necessary to reach a balance between the number of computational kernels and the amount of data needed by each one. This last value affects linearly the transference time and in a square mode the computation time. As shown in Figure 18, four nodes work in parallel. The transference time for each node is symbolized by TT Node *n* where *n* is the node number. If the amount of data loaded in each local memory allows an execution time long enough until new data is loaded, then the computing time shown in Figure 17 is actually equal to the total execution time. Time is calculated from formula (5), where *P* represent the amount of rows or columns in *M* × *P* multiplied by *P* × *N* matrix multiplication.

**Figure 19:**
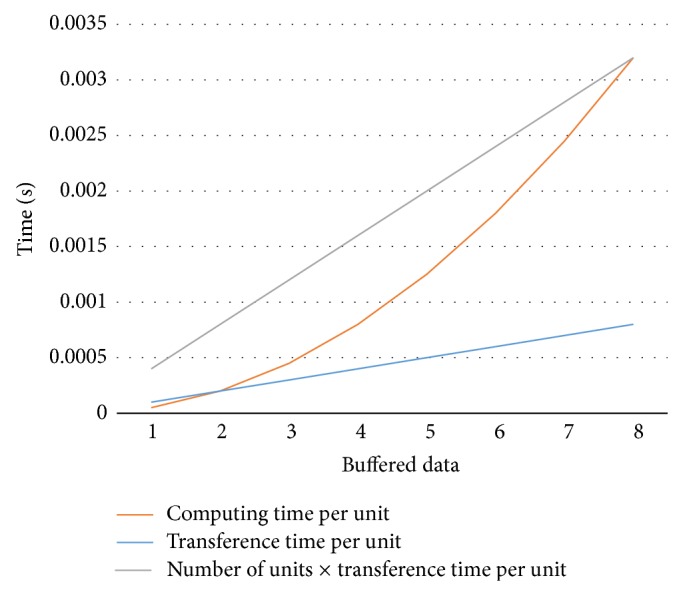
Computing versus transference time (5000 × 50004 4 × 4 16 bits).

Figure 19 represents the transference time for one computational kernel, the total transference time, and the computing time needed by each computational kernel, as a function of buffered data, for a 5000 × 5000 16-bit-wide element matrix multiplication, performed using 4 computational kernels of type 4 × 4. The buffered data indicates the amount of matrix elements of the matrix result that can be obtained.

It can be observed that the amount of data to be loaded in the local memory necessary to have a continuous execution time of each computational kernel is the necessary data to obtain 8 matrix elements of the result matrix:

(5) $$\begin{array}{c} \text{Tim}{\text{e}}_{\text{compute}}\geq \text{Tim}{\text{e}}_{\text{transference}}*N_{\text{kernels}},\\ N_{\text{cells}}^{2}*P\geq \frac{\text{Dat}{\text{a}}_{\text{width}}*P*2*\text{Kerne}{\text{l}}_{\text{size}}*N_{\text{cells}}*N_{\text{kernels}}}{\text{Bu}{\text{s}}_{\text{width}}},\\ N_{\text{cells}}\geq \frac{2*N_{\text{kernels}}*\text{Kerne}{\text{l}}_{\text{size}}*\text{Dat}{\text{a}}_{\text{width}}}{\text{Bu}{\text{s}}_{\text{width}}}. \end{array}$$

After all these considerations, the multiplication matrix was performed to measure the level of improvement that can be obtained using this approach. The computation time using this reconfigurable infrastructure with five 4 × 4 computational kernels was reduced from 1340 seconds using software until 16 seconds, which means an 83.75 times improvement.

## 7. Conclusion

In this paper, we introduce novel grid architecture and services for the integration of reconfigurable hardware (especially FPGAs) in the distributed high performance computing (HPC) world, so far dominated by multicore processors.

The principal contributions of this paper are on one side a computational model to integrate FPGAs at the same level of conventional processors, providing a transparent heterogeneous resources management and improving grid systems with the highest degree of efficiency that can be obtained for distributed computing applications using FPGAs and on the other side, a model for HPC applications development in order to exploit the benefits of this architecture. Besides, a set of services have been developed providing transparent application deployment including component location service and the mechanism for fast FPGA partial reconfiguration.

The infrastructure is made up of three layers: the lower layer is related to reconfigurable resources where user applications are instantiated, the second layer is for resource homogenization to facilitate application deployment, and the upper layer is for resource management issues. With this structure, resources are abstracted from the underlying platform in order to provide a homogeneous view for developers, facilitating migration between different computational node models.

This infrastructure was tested accelerating a 5000 × 5000 elements matrix multiplier application using two Virtex 5 FPGA with 8 reconfigurable areas in each one. Several analyses have been carried out to evaluate the viability of this work. Significant performance gains have been achieved and the obtained results encourage the use of this infrastructure for HPC application deployment. There are still some aspects that need to be object of study and are out of the scope of this paper, such as the incorporation of scheduling mechanisms for application deployment including partial reconfiguration scheduling, a dynamic sizing of reconfigurable areas, and tools to facilitate resource analysis according to application requirements, just to name a few of open problems.

## Figures and Tables

**Algorithm 1:**
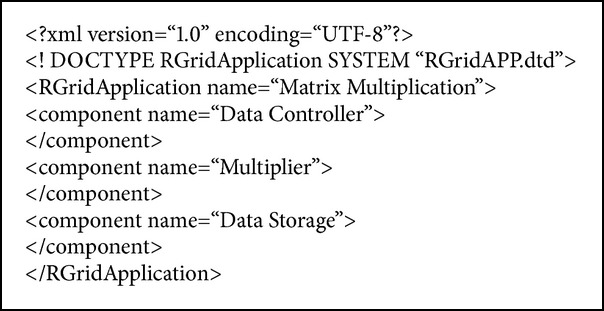
Example of an Application Descriptor.

**Algorithm 2:**
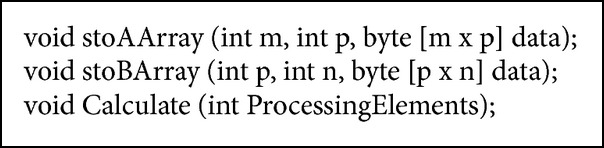
Data Controller Role Description.

**Algorithm 3:**
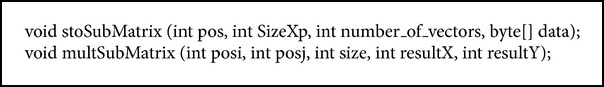
Processor Role Description.

**Algorithm 4:**
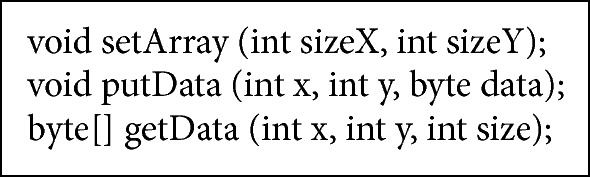
Storage Role Description.

**Table 1:** Platform services and their corresponding op. ID.

OP. ID	Service description
0	Component registration service: it is the component registration in local locator
1	Location service: it gives the address of an application component
2	Deployment service: it deploys the binary file which is sent in the parameter field
3	Component start service: it sends a signal to start a component
4	Stop service: it stops a component that is running
5	Node ID update service: it changes the node ID at node network level
6	Discovery message service: when this service is selected, the resource announcement message is in the parameter field
7	Free resources available description service: it describes the number of node free resources

**Table 2:** Resources used in communication mechanism in computational node.

Component	Slices	LUTs	FFs	Freq.
Network stack	1788	1380	2790	127.6 MHz
Adapters	624	643	981	186.4 MHz
Bus	284	210	335	220 MHz

**Table 3:** Logical resources used by deployment service.

Slices	LUTs	FFs	Frequency
376	631	755	234.9 MHz

**Table 4:** Time consumed during location process.

Location process	Time
C to A	230 nsec
C to B	720 *μ*sec
